# Clinical Implications of Nutritional Consultations and Oral Nutritional Supplementation Refusal in Head and Neck Cancer Patients During Chemoradiotherapy

**DOI:** 10.3390/nu18172765

**Published:** 2026-08-24

**Authors:** Aleksandra Krzywon, Anna Kotylak, Jolanta Mrochem-Kwarciak, Tomasz Rutkowski

**Affiliations:** 1 Department of Biostatistics and Bioinformatics, Maria Sklodowska-Curie National Research Institute of Oncology, Gliwice Branch, Wybrzeze Armii Krajowej 15, 44-102 Gliwice, Poland; 2 Department of Applied Informatics, Silesian University of Technology, Akademicka 16, 44-100 Gliwice, Poland; 3 I Radiation and Clinical Oncology Department, Maria Sklodowska-Curie National Research Institute of Oncology, Gliwice Branch, Wybrzeze Armii Krajowej 15, 44-102 Gliwice, Poland; anna.kotylak@gliwice.nio.gov.pl; 4 Analytics and Clinical Biochemistry Department, Maria Sklodowska-Curie National Research Institute of Oncology, Gliwice Branch, Wybrzeze Armii Krajowej 15, 44-102 Gliwice, Poland; jolanta.mrochem-kwarciak@gliwice.nio.gov.pl; 5 Clinical Trials Support Centre, Maria Sklodowska-Curie National Research Institute of Oncology, Gliwice Branch, Wybrzeze Armii Krajowej 15, 44-102 Gliwice, Poland; tomasz.rutkowski@gliwice.nio.gov.pl

**Keywords:** head and neck cancer, chemoradiotherapy, nutritional counseling, oral nutritional supplements, enteral nutrition

## Abstract

**Background/Objectives**: Nutritional support may improve the tolerance and effectiveness of definitive treatment in patients with head and neck cancer (HNC). The aim of this study was to investigate the potential clinical deterioration in patients who refuse nutritional consultations and oral nutritional supplementation during chemoradiotherapy (CHRT) for HNC. **Methods**: The study included 52 patients undergoing CHRT for HNC. Among these patients, 22 (42.3%) received nutritional counseling with oral nutritional supplementation (NCONS), while 30 (57.7%) patients refused NCONS (R-NCONS). The study aimed to investigate and compare the association of NCONS with weight loss, blood parameters, CHRT complications, and overall survival (OS) between the two groups. **Results**: At the end of CHRT, the R-NCONS group exhibited significantly higher CRP levels (18 mg/L vs. 4 mg/L; *p* = 0.013) and lower prealbumin levels (0.19 g/L vs. 0.27 g/L; *p* = 0.003) compared with NCONS group. Furthermore, R-NCONS patients experienced a higher rate of overall complications during CHRT (67% vs. 32%; *p* = 0.013), required enteral nutrition more frequently (20% vs. 0%; *p* = 0.033), and exhibited greater weight loss (−11% vs. −5%; *p* < 0.001). However, the 5-year OS did not differ significantly between the groups. **Conclusions**: The study demonstrates that while NCONS minimizes overall complications during CHRT, its refusal results in weight loss, elevated CRP and depleted prealbumin levels, and a more frequent need for enteral nutrition interventions.

## 1. Introduction

Head and neck cancer (HNC) is a group of cancers located in the upper aerodigestive tract, representing the fifth most common cancer worldwide. The main risk factors for HNC are tobacco use, alcohol consumption, and infection with human papillomavirus (HPV) or Epstein–Barr virus (EBV) [1,2,3,4]. Treatment consists of surgery, radiotherapy (RT), and chemotherapy (CHT) alone or in combination, according to the stage of the disease and the tumor site [5,6].

Malnutrition in cancer patients is defined as a deficiency of nutritional intake, resulting in tissue breakdown that leads to weight loss, as well as to impaired treatment tolerance and outcome [7,8,9]. Approximately 50% of cancer patients lose more than 10% of their body weight [10], and 10–20% of them die from malnutrition rather than from tumor progression [11]. This condition is largely unrecognized, underestimated, not prevented enough and undertreated in clinical practice [12]. It represents one of the most critical challenges in oncology, arising from both the tumor location and therapy-related side effects [8]. In patients with HNC, nutritional deficits are frequently observed due to dysphagia or digestive difficulties [13]. The side effects of therapy may additionally increase malnutrition [14]. To prevent malnutrition in this group of patients, the European Society for Clinical Nutrition and Metabolism (ESPEN) recommends individualized nutritional counseling with or without oral nutritional supplements and enteral nutrition (nasogastric or percutaneous tubes) if the patient exhibits treatment-related symptoms that impair oral intake [12].

The aim of this study was to evaluate the differences between patients with HNC who underwent chemoradiotherapy (CHRT) and received oral nutritional supplementation (NCONS) and those who refused it (R-NCONS), regarding body weight loss, blood-derived factors, treatment complications, and treatment outcomes.

## 2. Materials and Methods

### 2.1. Patient Characteristics and Data Collection

Between 2017 and 2018, 171 patients with HNC were treated at the Maria Sklodowska-Curie National Research Institute of Oncology, Gliwice Branch. Patients were treated using various oncological modalities—including induction CHT followed by CHRT or RT, as well as CHRT or RT alone—during which all participants were hospitalized and received nutritional support provided by a qualified dietitian. Out of the original 171 patients, a subset of 52 underwent CHRT. From this group, 22 adhered to the dietitian’s recommendations (NCONS), and 30 demonstrated low or no adherence to them (R-NCONS). NCONS consists of daily face-to-face nutritional consultations and oral nutritional supplementation (ONS) provided twice a day. The nutritional consultations involved prescribing hospital meals regular hospital food (90 to 120 g of protein/d and 2200 to 2500 kcal/d) with targeted texture modifications based on treatment-induced toxicities. The dietitian provided guidance on appropriate dietary intake and restricted items, such as spicy or hard foods and fresh fruits. Additionally, patient education focused on healthy eating principles, the clinical objectives and significance of oncology nutrition, post-treatment lifestyle modifications, dietary requirements, and the avoidance of alcohol consumption and smoking. ONS was administered twice daily (Nutridrink Protein, Nutricia; 125 mL containing 18 g of protein and 306 kcal). In the R-NCONS group, patients demonstrated non-compliance with the proposed nutritional modifications and dietary recommendations provided during consultations and failed to consume ONS. Specifically, depending on the individual patient, non-adherent behaviors included a total refusal to cooperate, falsification of reports regarding hospital food intake, consumption of fast food, reliance solely on dietary supplements while rejecting hospital meals, active alcohol consumption and smoking during treatment, and discarding hospital meals (90 to 120 g of protein/d and 2200 to 2500 kcal/d). During daily visits, the dietitian conducted a 24 h dietary recall to estimate the percentage of energy and protein requirements met by the patient. This information was subsequently communicated to the nursing staff, with daily nutritional progress and status documented exclusively within the dietitian’s internal records. Additionally, changes in body weight, as well as serum prealbumin (preALB) and albumin (ALB) concentrations, were evaluated on a weekly basis and logged within the same internal records. Except for ALB and pre-ALB, all other pre- and post-treatment blood samples were obtained within two weeks of treatment initiation and completion, respectively. Percutaneous endoscopic gastrostomy (PEG) tubes or nasogastric tubes were inserted when patients could not meet their nutritional needs and consented to enteral nutrition. The cohort in this study was derived from a database previously published in a different research context, and the majority of data from patients who refused NCONS were not analyzed [15]. Furthermore, data concerning the completion of CHRT, treatment interruptions, complications, and blood parameters were previously not investigated. Complications included pneumonia, cardiac events, bacterial infections, hepatotoxicity, nephrotoxicity, radiation dermatitis, phlebitis, and depressive episodes. Compliance with CHRT or RT was defined as the completion of all scheduled treatment cycles or fractions, even if temporary interruptions occurred due to complications. Nutritional Risk Score-2002 (NRS-2002) was used to assess nutritional risk before and after CHRT; a score of ≥3 indicated the need to initiate nutritional support [16].

### 2.2. Treatment

All patients underwent definitive treatment including concomitantly combined RT and CHT. RT doses ranged from 66 Gy in 33 fractions to 70 Gy in 35 fractions. CHT was platinum-based in all cases, given at a dose of 100 mg/m^2^ every 21 days or 40 mg/m^2^ every week concomitantly with RT.

### 2.3. Statistical Analysis

The basic characteristics of the included patients were summarized as frequencies and percentages for categorical variables and as median values with interquartile ranges (25% to 75%, IQR) for continuous variables. Continuous variables were compared between groups using Wilcoxon’s rank sum test. Categorical variables were compared between groups using Fisher’s exact test for 2 × 2 contingency tables, or the Fisher–Freeman–Halton test for larger, multi-column two-row tables. The Fisher–Freeman–Halton test was not applied to specific types of complications (which would require a 2 × 11 contingency table) because the high frequency of zero or single-digit cell counts would drastically reduce the statistical power of the test. Instead, Fisher’s exact test was performed separately for each individual complication type by collapsing the data into 2 × 2 tables. All individual complications were pooled into a single composite variable to perform Fisher’s exact test for overall complications. To examine the correlations between variables, Spearman correlation coefficients were calculated. Correlations were classified as follows: 0.0 ≤ |r| < 0.1 (negligible correlation), 0.1 ≤ |r| ≤ 0.39 (weak correlation), 0.4 ≤ |r| ≤ 0.69 (moderate correlation), 0.7 ≤ |r| ≤ 0.89 (strong correlation), and 0.9 ≤ r ≤ 1 (very strong correlation). Survival outcomes were estimated using the Kaplan–Meier method. To compare survival curves between two groups, the log-rank test with 95% confidence intervals was used. Overall survival (OS) was calculated from the date of end of treatment until the date of death or the last date of patient follow-up. The following packages were used: survival (version 3.8.6) [17], survminer (version 0.5.2) [18], and gtsummary (version 2.5.0) [19]. All computational analysis was performed in the R environment for statistical computing version 4.5.3 released on March 11, 2026 (R Foundation for Statistical Computing, Vienna, Austria, http://www.r-project.org). A two-sided *p*-value < 0.05 was considered statistically significant.

## 3. Results

There were no significant differences between these two groups regarding gender, age, T and N stage, tumor site, NRS-2002, ECOG performance status, and the Charlson Comorbidity Index (Table 1).

No differences between the R-NCONS and NCONS before treatment were present for body weight, BMI, ALB, preALB, CRP, Hgb, RBC, Retic, Hgb-Retic and PLT (Table 2).

At the end of CHRT, body weight (*p* = 0.027) and preALB level (*p*= 0.003) were significantly lower for the R-NCONS than for NCONS. The CRP level was higher for R-NCONS (*p* = 0.013). The percentage of weight loss was twice as high in the R-NCONS group compared to the NCONS group (<0.001) (Table 3). A strong negative correlation between CRP and preALB was observed in the R-NCONS group (r = −0.68, *p* < 0.001), but not in the NCONS group (r = −0.40, *p* = 0.068).

Table 4 shows a comparison of CHRT complications, completion of treatment and outcomes between the NCONS and R-NCONS. No significant differences were found in the severity of mucositis between groups (*p* = 0.5) at the end of CHRT. No differences were observed in the percentage of patients who completed RT (*p* = 1) or CHT (*p* = 0.12), according to breaks in RT (*p* = 0.4) or CHRT (*p* = 1.0) and length of hospital stay (*p* = 0.3). The R-NCONS received significantly more enteral nutrition than the NCONS (*p* = 0.033). Furthermore, the R-NCONS experienced more overall complications during CHRT (*p* = 0.013). Additionally, a significantly higher proportion of patients in the R-NCONS group had an NRS-2002 ≥ 3 (*p* = 0.02) at the end of treatment.

**Figure 1:**
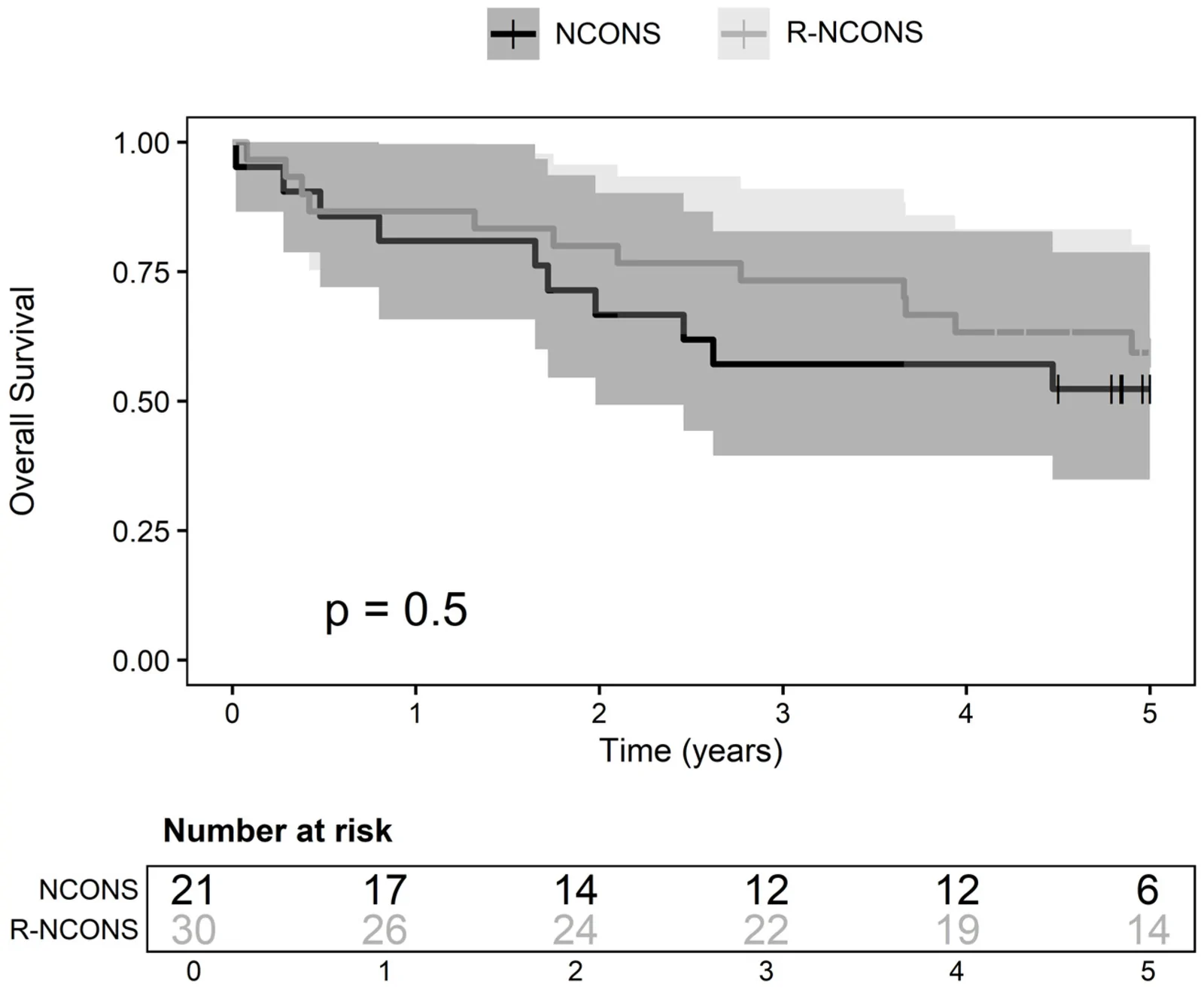
The Kaplan–Meier curve of overall survival in the NCONS and R-NCONS.

The 5-year OS rates were 59.4% (95% CI, 44.0–80.2) and 52.4% (95% CI, 34.8–78.8) for the R-NCONS and NCONS, respectively (*p* = 0.5) (Figure 1).

## 4. Discussion

The findings of this retrospective study indicate that NCONS refusal during CHRT was associated with greater weight loss, a higher incidence of overall complications, elevated CRP and depleted preALB levels, and an increased requirement for enteral nutrition interventions; however, overall survival (OS) did not differ between the NCONS and R-NCONS groups.

In clinical practice, weight loss is the primary parameter used to describe nutritional status [20]. This loss, mainly affecting fat-free mass, occurs in patients with HNC due to both the tumor itself and oncological interventions. Tumor location may obstruct the digestive tract, leading to difficulties with biting, chewing, or swallowing; furthermore, toxicity is typically exacerbated by RT, especially when combined with CHT [21]. Nutritional deficiencies have a negative impact on mortality, morbidity and quality of life [22]; therefore, nutritional support becomes a key element of supportive care in patients with HNC [23]. The European Society for Clinical Nutrition and Metabolism (ESPEN) recommends individualized nutritional counseling with the use of ONS as basic nutritional care for HNC patients [12]. The 2026 guidelines from the American Society for Parenteral and Enteral Nutrition (ASPEN) indicate that if HNC patients fail to maintain adequate oral intake during CHRT despite strategies like ONS, enteral nutrition is recommended. According to these guidelines, patients should achieve a target daily intake of ≥30 kcal/kg of energy and 1.2–1.5 g/kg of protein [24]. Our results showed that patients who refused NCONS lost significantly more weight than those in the NCONS group. This observation is consistent with the study by Jiang et al. [25], in which HNC patients, who received only general dietary advice without ONS, lost more weight compared to those who received daily nutritional advice with ONS. Furthermore, the HNC patients who received only general nutritional advice during RT [26,27] exhibited a significantly lower body weight compared with the group who received individualized nutritional counseling. In a randomized clinical trial [28], HNC patients were divided into an early nutritional intervention group, where ONS was initiated two weeks prior to treatment, and a control group, which received ONS only when oral intake became inadequate, based on dietitian recommendations. Patients in the intervention group experienced significantly less weight loss.

PreALB and CRP are both liver proteins and acute-phase inflammatory biomarkers [29]. PreALB is reduced during acute inflammation and decreased significantly more in the R-NCONS group than in the NCONS group at the end of CHRT. Conversely, CRP levels typically rise during inflammation. In line with this, we found a significant increase in CRP within the R-NCONS group at the end of CHRT. David et al. [30] showed that there is a negative correlation between CRP and preALB (r = −0.54, *p* < 0.001). Our results are consistent with these findings, indicating a strong negative correlation between CRP and preALB in the R-NCONS group. The greater decrease in preALB in the R-NCONS group compared to the NCONS group is consistent with a randomized trial showing that the initiation of ONS two weeks prior to treatment leads to better outcomes than receiving ONS only when recommended by a dietitian [28].

NCONS appears to be associated with a reduction in toxicity during CHRT (23% in the NCONS group vs. 67% in the R-NCONS group, *p* = 0.013). Our results are consistent with the findings of Leistra et al. [31], who reported that HNC patients receiving early individualized nutritional counseling post-surgery and prior to CHRT experienced a lower overall complication rate (including pneumonia, oral infection, and fistula) compared to those receiving general nutritional counseling.

The European Society for Clinical Nutrition and Metabolism (ESPEN) recommends enteral nutrition in the form of nasogastric or percutaneous tubes (e.g., percutaneous endoscopic gastrostomies (PEGs) prior to oncological treatment if there is a high risk of weight loss, as is the case for patients with HNC [12]. Paccagnella et al. [32] showed that, at the end of CHRT, 60.6% of HNC patients needed tube feeding. In the study by Arribas et al. [33], a nasogastric tube was required in 35% of HNC patients. In both studies, personalized nutritional counseling was performed, and ONS or enteral nutrition was used based on nutritional status. Our results showed that while 20% of patients in the R-NCONS group required enteral nutrition support, none of the patients in the NCONS group required such intervention. These findings suggest that daily nutritional counseling combined with ONS may be associated with maintaining body weight in HNC patients.

Unplanned treatment breaks in RT or CHRT significantly influence treatment outcomes. Gaps in therapy allow the regrowth of cancer cells and promote the proliferation of those who are resistant to CHT [34,35]. According to Giddings et al. [36], treatment breaks lasting more than a week were observed in up to 25% of patients (range: 12–28%). The main cause of such breaks during CHRT is mucositis [37]. We found that more than 80% of patients presented with at least grade 2 mucositis at the end of treatment in both the NCONS and R-NCONS groups; however, the incidence of treatment breaks did not differ between the groups. Paccagnella et al. [32] showed that the proportion of patients who completed CHRT was comparable between a weekly individualized NCONS group and a general nutritional counseling group. Jiang et al. [25] also found no statistical significance in CHRT completion rates between a group receiving weekly nutritional counseling with daily ONS (89%) and a group with weekly nutritional counseling without ONS (90%). This is consistent with our results, where 77% of patients completed CHRT in the NCONS group and 57% in the R-NCONS group.

Although nutritional support did not directly extend survival in the NCONS group compared to the R-NCONS group in this study, the existing literature, supported by our current findings, underscores numerous clinical benefits of its incorporation into oncological care. Firstly, the European Society for Clinical Nutrition and Metabolism (ESPEN) recommends combining nutritional counseling with oral nutritional supplements during CHRT to minimize therapy interruptions in patients with HNC or gastrointestinal cancers [38]. Secondly, patients who adhered to NCONS experienced fewer overall complications during oncological treatment, as demonstrated in our study. Thirdly, as demonstrated by randomized controlled trials [39], NCONS significantly improves the quality of life of patients undergoing RT compared to nutritional counseling alone. Furthermore, because eating is deeply linked to social interactions, and HNC patients frequently experience difficulties dining around others post-treatment [40,41], preserving this function during therapy is of paramount importance. In addition, inadequate food intake exacerbates cancer cachexia, a debilitating condition to which HNC patients are highly susceptible [42]. This is underscored by the fact that these patients commonly lose more than 5% of their total body weight over the course of CHRT [43,44]. Finally, well-nourished cancer patients experience shorter hospital stays, which significantly reduces overall healthcare costs [42]. According to a recent meta-analysis [45], severe (grade ≥ 3) CHRT-induced mucositis could not be effectively prevented by NCONS alone. The fact that we did not observe a significant effect of NCONS on survival within this patient subgroup may be due to the small sample size; therefore, these results should be interpreted with caution.

The limitations of this study include its retrospective nature, single-center design, and relatively small patient cohort. Furthermore, most patients in the R-NCONS group were of low socioeconomic status and had limited educational backgrounds. Most exhibited alcohol and nicotine dependence, demonstrated poor self-care behaviors, and presented substantial management challenges during treatment. They were largely non-compliant with recommendations from dietitians, dental hygienists, and nursing staff. Specifically, ongoing alcohol consumption and the unsupervised use of dietary supplements of unknown efficacy or interaction potential introduced significant confounding variables that may have impacted treatment effectiveness and clinical outcomes. Conversely, the fact that the R-NCONS group received enteral nutrition represents another important variable, as it may have inadvertently improved their clinical outcomes and counteracted some of these adverse lifestyle factors. Despite these confounding factors, we believe that our findings remain highly relevant, as they offer valuable insight into the outcomes of patients who do not receive nutritional support. Currently, establishing such a control group in prospective or randomized controlled trials is impossible due to ethical constraints. Because ESPEN guidelines strongly recommend dietary consultations and oral nutritional supplements, evaluating this unique cohort—despite its numerous confounding variables—represents a major strength of this study.

## 5. Conclusions

Our study indicates that refusal to use NCONS may be associated with an increased incidence of CHRT-induced overall complications, severe weight loss, elevated CRP, depleted prealbumin, and greater reliance on enteral nutrition.

## Figures and Tables

**Table 1:** Patient, tumor and treatment characteristics.

Variable	NCONS, N = 22	R-NCONS, N = 30	*p*-Value
**Gender (%)**			0.056
female	4 (18%)	13 (43%)	
male	18 (82%)	17 (57%)	
**Age** median [IQR]	56 [53–60]	60 [54–63]	0.2
**T stage (%)**			0.4
T (0–1)	12 (55%)	20 (67%)	
T (3–4)	10 (45%)	10 (33%)	
**N stage (%)**			0.8
N (0–1)	9 (41%)	11 (37%)	
N (2–3)	13 (59%)	19 (63%)	
**Treatment** (**%**)			
CHRT	22 (100%)	30 (100%)	1.0
**Tumor site (%)**			0.8
oral cavity	6 (27%)	7 (23%)	
oropharynx	10 (45%)	11 (37%)	
nasopharynx	2 (9.1%)	2 (6.7%)	
hypopharynx	1 (4.5%)	4 (13%)	
larynx	1 (4.5%)	4 (13%)	
other	2 (9.1%)	2 (6.7%)	
**NRS-2002 (%)**			1
<3	22 (100%)	29 (96.7%)	
≥3	0 (0%)	1 (3.3%)	
**ECOG performance status (%)**			0.7
0	13 (59%)	16 (53%)	
1	9 (41%)	14 (47%)	
**Charlson Comorbidity Index (%)**			0.8
0	0 (0%)	2 (6.7%)	
2	1 (4.5%)	1 (3.3%)	
3–4	9 (41%)	12 (40%)	
>4	4 (18%)	3 (10%)	
unknown	8 (36%)	12 (40%)	

Abbreviations: NRS—nutritional risk score.

**Table 2:** Body weight, BMI and laboratory parameters before treatment.

Variable	NCONS, N = 22	R-NCONS, N = 30	*p*-Value
**Body weight (kg)** median [IQR]	76 [68–88]	74 [67–82]	0.5
**BMI (kg/m^2^)** median [IQR]	25.7 [23.0–27.9]	26.2 [22.6–29.1]	0.7
**ALB (g/L)** median [IQR]	41.5 [40.0–42.9]	41.0 [39.0–44.0]	0.7
**preALB (g/L)** median [IQR]	0.29 [0.26–0.31]	0.25 [0.23–0.29]	0.060
**CRP (mg/L)** median [IQR]	1.1 [0.5–5.3]	2.5 [1.0–10.0]	0.2
**Hgb (g/dL)** median [IQR]	14 [13–14.8]	13.70 [13.4–14.6]	>0.9
**RBC (10^12^/L)** median [IQR]	4.9 [4.0–5.0]	4.7 [4.4–5]	0.6
**Retic (10^9^/L)** median [IQR]	56 [25–82]	61 [38–84]	0.3
**Hgb-Retic (pg)** median [IQR]	33.6 [31.3–35.8]	33.4 [32.7–34.8]	>0.9
**PLT (10^9^/L)** median [IQR]	267 [235, 291]	253 [216, 301]	0.6

Abbreviations: IQR—interquartile range, BMI—body mass index, CRP—c-reactive protein, Hgb—hemoglobin, RBC—red blood cell, Retic—reticulocytes, Hgb-Retic—reticulocyte hemoglobin equivalent, PLT—platelet count, ALB—albumin, preALB—prealbumin.

**Table 3:** Body weight, BMI, and laboratory parameters after treatment.

Variable	NCONS, N = 22	R-NCONS, N = 30	*p*-Value
**Body weight (kg)** median [IQR]	74 [66–80]	66 [59–74]	0.027
Change in weight ** [%]	−5 (−7, −1)	−11 (−15, −8)	<0.001
**BMI (kg/m^2^)** median [IQR]	24.4 [22.3–26.1]	23.9 [19.8–26.2]	0.4
**ALB (g/L)** median [IQR]	38.0 [35.0–39.8]	36.0 [34.0–38.8]	0.14
**preALB (g/L)** median [IQR]	0.27 [0.24–0.33]	0.19 [0.14–0.28]	0.003
**CRP (mg/L)** median [IQR]	4 [1–13]	18 [8–34]	0.013
**Hgb (g/dL)** median [IQR]	12.6 [11.4–13.1]	11.6 [10.5–13.2]	0.3
**RBC (10^12^/L)** median [IQR]	4.2 [3.7–4.5]	3.9 [3.5–4.4]	0.4
**Retic (10^9^/L)** median [IQR]	64 [49–97]	78 [52–105]	0.5
**Hgb-Retic (pg)** median [IQR]	34.5 [33.9–35.8]	33.6 [31.3–35.7]	0.3
**PLT (10^9^/L)** median [IQR]	232 [184–259]	273 [173–337]	0.2

Abbreviations: IQR—interquartile range, BMI—body mass index, CRP—c-reactive protein, Hgb—hemoglobin, RBC—red blood cell, Retic—reticulocytes, Hgb-Retic—reticulocyte hemoglobin equivalent, PLT—platelet count, ALB—albumin, preALB—prealbumin, **—percent change = (end of treatment parameter -beginning of treatment parameter)/end of treatment parameter × 100%).

**Table 4:** Comparison of CHRT-related complications, completion of treatment and outcomes for NCONS and R-NCONS.

Variable	NCONS, N = 22	R-NCONS, N = 30	*p*-Value
**Mucositis (grade, %)**			0.5
2	4 (18%)	4 (13%)	
3	18 (82%)	24 (80%)	
4	0 (0%)	2 (6.7%)	
**Enteral nutrition (%)**	0 (0%)	6 (20%)	0.033
**Overall complications during CHRT (%)**	7 (32%)	20 (67%)	0.013
**Types of complications during CHRT (%)**			
hematological toxicity	6 (27%)	4 (13%)	0.29
hematological toxicity and pneumonia	0 (0%)	1 (3.3%)	1.0
hematological toxicity and bacterial infection	0 (0%)	4 (13%)	0.13
hematological toxicity and other	0 (0%)	1 (3.3%)	1.0
pneumonia	0 (0%)	2 (6.7%)	0.5
cardiac complications and bacterial infection	0 (0%)	1 (3.3%)	1.0
bacterial infection	0 (0%)	3 (10%)	0.25
hepatotoxicity	0 (0%)	1 (3.3%)	1.0
nephrotoxicity	0 (0%)	1 (3.3%)	1.0
other (skin reaction IV, phlebitis, depressive episodes)	1 (4.5%)	1 (3.3%)	1.0
bacterial infection and other (skin reaction IV, phlebitis, depressive episodes)	0 (0%)	1 (3.3%)	1.0
**RT compliance (%)**	22 (100%)	30 (100%)	1.0
**CHT compliance (%)**	17 (77%)	17 (57%)	0.12
**RT interruption (%)**	2 (9.1%)	6 (20%)	0.4
**CHT interruption (%)**	1 (4.5%)	2 (6.7%)	1.0
**Length of hospital stay (days) median [IQR]**	55 (50–59)	56 (53–62)	0.3
**NRS-2002 (%)**			
<3	21 (95.5%)	20 (66.7%)	0.02
≥3	1 (4.5%)	10 (33.3%)	

Abbreviations: RT—radiotherapy; CHT—chemotherapy; CHRT—chemoradiotherapy.

## Data Availability

The datasets generated and/or analyzed during the current study are available from the corresponding author on reasonable request.

## References

[1] Sanderson R.J., Ironside J.A. (2002). Squamous cell carcinomas of the head and neck. BMJ.

[2] Argiris A., Karamouzis M.V., Raben D., Ferris R.L. (2008). Head and neck cancer. Lancet.

[3] Chow L.Q.M. (2020). Head and Neck Cancer. N. Engl. J. Med..

[4] Cohen N., Fedewa S., Chen A.Y. (2018). Epidemiology and Demographics of the Head and Neck Cancer Population. Oral Maxillofac. Surg. Clin. N. Am..

[5] Brewczyński A., Jabłońska B., Mrowiec S., Składowski K., Rutkowski T. (2020). Nutritional Support in Head and Neck Radiotherapy Patients Considering HPV Status. Nutrients.

[6] Maciejewski B., Gabrys D., Rembak-Szynkiewicz J., Napieralska A., Stapor-Fudzinska M. (2023). Have innovations in radiotherapy for head and neck cancer improved the curability of the disease?. Nowotw. J. Oncol..

[7] Bossi P., De Luca R., Ciani O., D’Angelo E., Caccialanza R. (2022). Malnutrition management in oncology: An expert view on controversial issues and future perspectives. Front. Oncol..

[8] Muscaritoli M., Lucia S., Farcomeni A., Lorusso V., Saracino V., Barone C., Plastino F., Gori S., Magarotto R., Carteni G. (2017). Prevalence of malnutrition in patients at first medical oncology visit: The PreMiO study. Oncotarget.

[9] Santarpia L., Contaldo F., Pasanisi F. (2011). Nutritional screening and early treatment of malnutrition in cancer patients. J. Cachexia Sarcopenia Muscle.

[10] Argilés J.M. (2005). Cancer-associated malnutrition. Eur. J. Oncol. Nurs..

[11] Beirer A. (2021). Malnutrition and cancer, diagnosis and treatment. Memo.

[12] Muscaritoli M., Arends J., Bachmann P., Baracos V., Barthelemy N., Bertz H., Bozzetti F., Hütterer E., Isenring E., Kaasa S. (2021). ESPEN practical guideline: Clinical Nutrition in cancer. Clin. Nutr..

[13] Martinovic D., Tokic D., Puizina Mladinic E., Usljebrka M., Kadic S., Lesin A., Vilovic M., Lupi-Ferandin S., Ercegovic S., Kumric M. (2023). Nutritional Management of Patients with Head and Neck Cancer-A Comprehensive Review. Nutrients.

[14] Milliron B.J., Packel L., Dychtwald D., Klobodu C., Pontiggia L., Ogbogu O., Barksdale B., Deutsch J. (2022). When Eating Becomes Torturous: Understanding Nutrition-Related Cancer Treatment Side Effects among Individuals with Cancer and Their Caregivers. Nutrients.

[15] Krzywon A., Kotylak A., Cortez A.J., Mrochem-Kwarciak J., Składowski K., Rutkowski T. (2023). Influence of nutritional counseling on treatment results in patients with head and neck cancers. Nutrition.

[16] Kondrup J., Rasmussen H.H., Hamberg O., Stanga Z., Ad Hoc ESPEN Working Group (2003). Nutritional risk screening (NRS 2002): A new method based on an analysis of controlled clinical trials. Clin. Nutr..

[17] Therneau T.M. (2026). A Package for Survival Analysis in R.

[18] Kassambara A., Kosinski M., Biecek P. (2026). Survminer: Drawing Survival Curves Using ggplot2.

[19] Sjoberg D.D., Whiting K., Curry M., Lavery J.A., Larmarange J. (2021). Reproducible summary tables with the gtsummary package. R J..

[20] van Bokhorst-de van der Schueren M.A., van Leeuwen P.A., Sauerwein H.P., Kuik D.J., Snow G.B., Quak J.J. (1997). Assessment of malnutrition parameters in head and neck cancer and their relation to postoperative complications. Head Neck.

[21] Ottosson S., Zackrisson B., Kjellén E., Nilsson P., Laurell G. (2013). Weight loss in patients with head and neck cancer during and after conventional and accelerated radiotherapy. Acta Oncol..

[22] Chasen M.R., Bhargava R. (2009). A descriptive review of the factors contributing to nutritional compromise in patients with head and neck cancer. Support. Care Cancer.

[23] Talwar B., Donnelly R., Skelly R., Donaldson M. (2016). Nutritional management in head and neck cancer: United Kingdom National Multidisciplinary Guidelines. J. Laryngol. Otol..

[24] Kiss N., Findlay M., Frowen J., Lewis W.E., Mills J., Singh A.K., Church D.D., Mey J.T., Peterson S., Aguzzi K. (2026). Guidelines for nutrition in adults with head and neck cancer: The American Society for Parenteral and Enteral Nutrition. JPEN J. Parenter. Enter. Nutr..

[25] Jiang W., Ding H., Li W., Ling Y., Hu C., Shen C. (2018). Benefits of Oral Nutritional Supplements in Patients with Locally Advanced Nasopharyngeal Cancer during Concurrent Chemoradiotherapy: An Exploratory Prospective Randomized Trial. Nutr. Cancer.

[26] Isenring E.A., Capra S., Bauer J.D. (2004). Nutrition intervention is beneficial in oncology outpatients receiving radiotherapy to the gastrointestinal or head and neck area. Br. J. Cancer.

[27] van den Berg M.G., Rasmussen-Conrad E.L., Wei K.H., Lintz-Luidens H., Kaanders J.H., Merkx M.A. (2010). Comparison of the effect of individual dietary counselling and of standard nutritional care on weight loss in patients with head and neck cancer undergoing radiotherapy. Br. J. Nutr..

[28] Jiang W., Zhang H., Dou S., He Y., Zhu G., Li R. (2025). Effectiveness of Early Oral Nutritional Supplementation in Preventing Weight Loss in Head and Neck Cancer Patients Undergoing Postoperative Radiotherapy or Chemoradiotherapy: A Prospective Randomized Controlled Trial. J. Am. Nutr. Assoc..

[29] Ranasinghe R.N., Biswas M., Vincent R.P. (2022). Prealbumin: The clinical utility and analytical methodologies. Ann. Clin. Biochem..

[30] Davis C.J., Sowa D., Keim K.S., Kinnare K., Peterson S. (2012). The use of prealbumin and C-reactive protein for monitoring nutrition support in adult patients receiving enteral nutrition in an urban medical center. JPEN J. Parenter. Enter. Nutr..

[31] Leistra E., Eerenstein S.E., van Aken L.H., Jansen F., de van der Schueren M.A., Twisk J.W., Visser M., Langius J.A. (2015). Effect of Early Individualized Dietary Counseling on Weight Loss, Complications, and Length of Hospital Stay in Patients with Head and Neck Cancer: A Comparative Study. Nutr. Cancer.

[32] Paccagnella A., Morello M., Da Mosto M.C., Baruffi C., Marcon M.L., Gava A., Baggio V., Lamon S., Babare R., Rosti G. (2010). Early nutritional intervention improves treatment tolerance and outcomes in head and neck cancer patients undergoing concurrent chemoradiotherapy. Support. Care Cancer.

[33] Arribas L., Hurtós L., Taberna M., Peiró I., Vilajosana E., Lozano A., Vazquez S., Mesia R., Virgili N. (2017). Nutritional changes in patients with locally advanced head and neck cancer during treatment. Oral Oncol..

[34] Rosenthal D.I. (2007). Consequences of mucositis-induced treatment breaks and dose reductions on head and neck cancer treatment outcomes. J. Support. Oncol..

[35] Patel S., Rich B.J., Schumacher L.D., Sargi Z.B., Masforroll M., Washington C., Kwon D., Rueda-Lara M.A., Freedman L.M., Samuels S.E. (2023). ED visits, hospital admissions and treatment breaks in head/neck cancer patients undergoing radiotherapy. Front. Oncol..

[36] Giddings A. (2010). Treatment Interruptions in Radiation Therapy for Head-and-Neck Cancer: Rates and Causes. J. Med. Imaging Radiat. Sci..

[37] Russo G., Haddad R., Posner M., Machtay M. (2008). Radiation treatment breaks and ulcerative mucositis in head and neck cancer. Oncologist.

[38] Arends J., Bodoky G., Bozzetti F., Fearon K., Muscaritoli M., Selga G., van Bokhorst-de van der Schueren M.A., von Meyenfeldt M., Zürcher G., DGEM (German Society for Nutritional Medicine) (2006). ESPEN Guidelines on Enteral Nutrition: Non-surgical oncology. Clin. Nutr..

[39] Cereda E., Cappello S., Colombo S., Klersy C., Imarisio I., Turri A., Caraccia M., Borioli V., Monaco T., Benazzo M. (2018). Nutritional counseling with or without systematic use of oral nutritional supplements in head and neck cancer patients undergoing radiotherapy. Radiother. Oncol..

[40] Dornan M., Semple C., Moorhead A. (2022). Experiences and perceptions of social eating for patients living with and beyond head and neck cancer: A qualitative study. Support. Care Cancer.

[41] Dornan D.M., Semple C.J., Moorhead A. (2024). Eating with Others’: Planning, developing and optimising a self-management intervention to promote social eating for patients living with and beyond head and neck cancer. Support. Care Cancer.

[42] Schuetz P., Sulo S., Walzer S., Krenberger S., Brunton C. (2022). Nutritional support during the hospital stay is cost-effective for preventing adverse outcomes in patients with cancer. Front. Oncol..

[43] Abu Zaid Z., Kay Neoh M., Mat Daud Z.A., Md Yusop N.B., Ibrahim Z., Abdul Rahman Z., Jamhuri N., Abdul Azim A.Z. (2022). Weight Loss in Post-Chemoradiotherapy Head and Neck Cancer Patients. Nutrients.

[44] Lin H.Y., Feng H.C., Wang Y.J. (2025). Trajectory of weight loss and nutritional decline in head and neck cancer patients undergoing concurrent chemoradiotherapy: A longitudinal observational study. Support. Care Cancer.

[45] Krzywon A., Kotylak A., Rutkowski T. (2025). Does nutritional support prevent severe mucositis in patients with head and neck cancer treated with chemoradiotherapy? A systematic review and meta-analysis. Clin. Nutr. ESPEN.

